# Modeling Cash Plus Other Psychosocial and Structural Interventions to Prevent HIV Among Adolescent Girls and Young Women in South Africa (HPTN 068)

**DOI:** 10.1007/s10461-021-03158-3

**Published:** 2021-01-21

**Authors:** Marie C. D. Stoner, Jessie K. Edwards, Daniel Westreich, Kelly Kilburn, Jennifer Ahern, Sheri A. Lippman, F. Xavier Gómez-Olivé, Kathleen Kahn, Audrey Pettifor

**Affiliations:** 1 Women’s Global Health Imperative, RTI International, Berkeley, CA USA; 2 Carolina Population Center, University of North Carolina, Chapel Hill, NC USA; 3 Department of Epidemiology, University of North Carolina, Chapel Hill, NY USA; 4 MRC/Wits Rural Public Health and Health Transitions Research Unit (Agincourt), School of Public Health, Faculty of Health Sciences, University of the Witwatersrand, Johannesburg, South Africa; 5 INDEPTH Network, Accra, Ghana; 6 Epidemiology and Global Health Unit, Department of Public Health and Clinical Medicine, Umeå University, Umeå, Sweden; 7 Department of Medicine, University of California, San Francisco, USA; 8 Division of Epidemiology & Biostatistics, School of Public Health, University of California, Berkeley, USA; 9 Duke Global Health Institute, Duke University, Durham, NC USA

**Keywords:** Adolescent girls, Young women, HIV prevention, Cash transfer, Social determinants, Causal inference

## Abstract

Poverty alleviation programs can reduce HIV incidence but may have greater impacts when combined with other psychosocial interventions. We modeled the change in HIV incidence among South African adolescent girls and young women (AGYW) associated with combining a cash transfer (the South African Child Support Grant (CSG)) with other structural and behavioral interventions. We modeled observational data from the HPTN 068 study where 2328 HIV negative AGYW (13–20 years) were followed for 4 years. In a Monte Carlo simulation based on this cohort (N = 10,000), CSG receipt was not independently associated with HIV incidence. Providing the CSG combined with increasing caregiver care and reducing adolescent depression had the largest reduction in HIV incidence with the fewest number of combined interventions (RD − 3.0%; (95% CI − 5.1%, − 0.9%). Combining a monthly grant with interventions to increase caregiver care and reduce adolescent depression could substantially reduce HIV incidence above the provision of cash alone.

## Introduction

Cash transfer programs, where households or individuals receive cash payments, have been shown to mitigate the social determinants of HIV risk such as poverty and lack of education and to improve psychosocial outcomes including anxiety, self-esteem and hope for the future [1]. These programs are usually national government cash transfer or “social protection” programs provided to poor and vulnerable households to alleviate poverty and improve health outcomes. For example, the Child Support Grant (CSG) in South Africa is available to relatively low-income households to help parents with the costs of basic needs for their children without restrictions on how the money should be spent. Given the success of these programs in improving school attendance and increasing household expenditures, cash payments have increasingly been studied as a strategy to prevent HIV infection. The theory behind the use of these programs in HIV prevention is that improving the underlying structural factors related to HIV risk such as socioeconomic conditions and school attendance will reduce risky behaviors and therefore prevent HIV [2, 3]. Yet, most studies that have assessed the impact of cash transfer programs on HIV risk have shown mixed results among adolescent girls and young women (AGYW), a population with a relatively high incidence of HIV infection [3–6].

Four studies have evaluated the impact of cash transfer interventions on HIV biomarkers among AGYW with mixed results. These interventions have provided cash to individual girls and their households either unconditionally or conditional on a behavior such as attending school. A cash transfer intervention to AGYW and their families to stay in school in Malawi reduced HIV prevalence (OR 0.36; 95% CI 0.14, 0.91) [4] and a recent intervention that provided incentives to AGYW to enroll and attend school in eSwatini reduced HIV incidence among those who received the incentives (OR 0.77; 95% CI 0.60–0.98) [7]. Conversely, a third study in South Africa had too few HIV infections to assess impact [6] and a fourth study, from which these data originate, found no impact on HIV incidence among AGYW with a cash transfer intervention that was conditional on attending 80% of school days [5]. A larger number of studies have evaluated the effects of cash transfer programs on sexual behaviors among AGYW [8–13]. In particular, six of the eight studies of government grant programs that have evaluated impacts on sexual debut in adolescents found a delay in sexual initiative or a reduction in sexual onset, highlighting the potential of cash interventions for this age group [8–13].

In addition, recent evidence has shown that cash transfer programs might be more effective when combined with other interventions to provide psychosocial resources in addition to economic resources. Poverty is a social determinant of health that affects risk of HIV through complex pathways that intersect with other multilevel social factors. For example, one study in South Africa found that receipt of the government CSG reduced incidence of HIV-risk behaviors for girls aged 12–18 years (OR 0.63, p = 0.02) but the reduction was larger when combining the CSG with teacher or caregiver support (as measured by a positive parenting scale including praise and warmth) (OR 0.55; 95% CI 0.35–0.85, P.0.01;) [14, 15] . Still, only this one study has evaluated “cash plus” strategies for HIV prevention and the study examined associations with sexual behaviors, but not with HIV incidence [14, 15] . Several additional cash plus care studies are currently ongoing but few are assessing impact on HIV incidence [16–23]. Among those that are examining incident infections [16, 19], these studies are limited to testing only select combinations (e.g. savings-led economic empowerment plus a family strengthening component) because of the high cost and large sample sizes required to implement randomized trials of multicomponent interventions.

The theory behind why these combined cash plus care interventions may be more effective is based on three main points [24]. First, sexual behaviors and HIV incidence among AGYW is related to multilevel and intersecting social factors at the level of the community, household, peers, and partner [25–28]. For example, economic instability is a driver of transactional sex, but is also associated with increased exposure to violence through a partner [29–32]. Second, the causal mechanisms by which different behaviors increase HIV-incidence may vary and intervening within more than one pathway could further reduce risk of HIV [24]. For example, age-disparate sex is increased by low school attendance [33], whereas unprotected sex is increased by power dynamics within a relationship [34, 35]. Third, adversities accumulate throughout childhood and adolescence to increase risk of sexual transmitted infections more than any single adversity [14, 24, 36]. Thus, combination social protection has the potential to maximize HIV prevention impacts by ameliorating simultaneous risks across the life course within these intersecting areas.

We used data from AGYW in rural South Africa to explore the potential effects of different combinations of receipt of a government CSG plus increasing school attendance, reducing intimate partner violence (IPV), reducing adolescent depression, and increasing perceptions that a parent/caregiver cares on incident HIV infection. School attendance [37], intimate partner violence (IPV) [38], and depression [39] have all been associated with incident HIV infection in prior analyses of these data from the HIV Prevention Trial Network 068 study (HPTN 068). In addition, we examined if reductions in HIV incidence seen with the CSG plus other psychosocial and structural interventions were further increased or modified by receipt of the conditional cash transfer (CCT) intervention that was tested in the original HPTN 068 trial. We examined the potential effects of both the CSG and CCT but focus primarily on the CSG because prior studies have examined the CSG [14] and because the CCT did not have an impact in the initial trial, which may have been because so many households (~ 80%) were already receiving a government grant to alleviate poverty (the CSG) [5].

## Methods

### Data

We analyzed data from the main trial period of the HPTN 068 study from 2011 to 2015. HPTN 068 was a randomized trial of an intervention to provide cash to AGYW and their households, conditional on 80% school attendance, as a way to prevent HIV acquisition in AGYW [5, 40]. The study enrolled 2533 AGYW aged 13–20 years who were in school, were not pregnant or married, had a parent/guardian in the household and were living in the rural Bushbuckridge sub-district of Mpumalanga province, South Africa. The area is the site of the Agincourt Health and Socio-Demographic Surveillance System (HDSS) with high levels of poverty, migration for work, poor infrastructure (e.g. unpaved roads) and a high prevalence of HIV [41].

During the trial, young women were followed for up to 3 years until study completion or graduation from high school, whichever came first. AGYW were visited annually and each visit included an Audio Computer-Assisted Self-Interview (ACASI) with the AGYW and her parent/guardian, and a test for HIV and Herpes Simplex Virus Type 2 (HSV-2). Our analysis includes only AGYW who had at least one follow-up visit and were HIV negative at enrollment.

### Variables

The outcome of incident HIV infection was defined as new cases of HIV identified over the study period. New cases were defined as two reactive rapid tests with a confirmatory Western Blot. The exposures of interest were time-varying household receipt of a government CSG, baseline adolescent depression, baseline physical intimate partner violence, time-varying high attendance in school, time-varying caregiver care and randomization to the cash transfer treatment arm at baseline. Receipt of the CSG was defined as report from the parent/guardian at each visit that at least one member of the household was receiving a CSG from the South African Government. Adolescent depression was a binary variable defined as having a Children’s Depression Inventory score of greater than or equal to seven at enrollment [42]. Physical intimate partner violence was a binary variable defined as having ever experienced any physical intimate partner violence (IPV) by a partner at enrollment based on the WHO global questionnaire [5, 43]. High attendance in school was a time-varying variable defined at each visit as attending ≥ 80% of school days in the months between study visits and was dichotomized as high attendance versus low attendance (< 80% school days), based on the condition used to receive cash in the original trial [5, 37]. The attendance variable was constructed from high school attendance registers from schools where the young women were enrolled. Perception that a parent/guardian cares was a time-varying binary variable constructed based on the question asked at each visit of the young woman “How much do you feel that [parent/guardian] cares for you.” Caregiver care was defined as answering that the parent/guardian cares “a lot” or “somewhat” compared to answering “not at all”. Receipt of the CCT intervention was defined as being randomized to the intervention arm of the trial at enrollment. Most measures were adapted from questionnaires that have been used previously in South Africa.

Covariates were selected by drawing a diagram of relationships between joint exposures, confounders, and HIV infection, and each individual exposure-outcome relationship. All confounders were selected based on prior literature indicating the importance of these variables. Confounders included time interval, age at enrollment, time-varying socioeconomic status (SES) (asset quartiles), and time-varying orphan status (one or both parents died when the young woman was < 18 years). We did not adjust for other grants in the household but did adjust for SES to account for overall household SES.

### Statistical Analysis

We modeled potential interventions on each exposure using the g-formula. The g-formula can be used to model how interventions might hypothetically reduce HIV risk similar to other forms of simulation modeling [44]. First, we estimated the average association between each exposure and HIV incidence by comparing HIV incidence if all girls had an exposure versus if no girls had the exposure. For example, the risk of HIV if all girls were in a household that received a CSG at each visit versus if all girls were in households that did not receive a CSG. Second, we estimated the HIV risk given the observed (real) distribution of each risk factor compared to a population in which all girls had risks removed. For example, the risk of HIV if all girls were in a household that received a CSG at each visit versus if 79.7% (observed) were in a household that received the CSG. Third, we modeled potential interventions by changing the CSG exposure in combination with reducing other risk factors and receipt of the CCT. We estimated the potential effect of each combination of factors on incident HIV infection compared with the risk of HIV infection that was observed in the data set with the observed (real) distribution of each risk factor. Lastly, we examined combinations stratified by CCT intervention arm. To assess the causal assumption of positivity, we checked the data to ensure that we had individuals exposed and unexposed in all covariate strata for the combinations. Under a set of identifiability assumptions, the associations estimated in this manuscript can be interpreted as causal effects, but we refer to these estimates as “associations” or “potential effects” because of possible violations to these assumptions. More discussion of the possible violations of these assumptions is included in the discussion section.

To implement the g-formula, we (i) parametrically modeled probabilities of the exposures, time-varying confounders and the outcome at each time point, conditional on exposure history and covariates in the observed data [45–52]. We used pooled logistic models for binary variables and linear regression models for continuous variables. We modeled time points until infection or graduation from high school (over grade 12), as this is when AGYW would have exited the trial. We then (ii) drew a Monte Carlo sample of 10,000 participants drawn with replacement from the observed data. We (iii) used the conditional probabilities estimated in (i) to predict risk of HIV by time *t*. We then (iv) compared our predicted risk under no intervention on the exposure (i.e., under the “natural course” [44]) with the observed data to assess the fit of the parametric models [44, 45]. We also compared the natural course (simulated) with the observed data for exposures, and time-varying confounders to make sure that the simulated cohort accurately represented the observed data. Finally, we (v) estimated risk under each intervention scenario by setting the values of factors individually and in combination in the simulated cohort. We computed 95% CIs by repeating the above steps (i) through (vi) on 500 nonparametric bootstrap resamples of the original data.

We compared risk of HIV at the end of the study period (4 years) under each intervention using risk differences and risk ratios calculated using the complement of the extended Kaplan Meir estimator [45]. Interventions that were assessed included the CSG and all combinations of increasing caregiver care, decreasing low school attendance, decreasing IPV and decreasing adolescent depression. An intervention on the CCT was added to the prior combinations that had the largest reductions in HIV incidence. Two-way interaction terms were included between all potential exposures in the outcome model to estimate joint effects of these exposures. We also assessed interaction between the CSG and each exposure by calculating the interaction contrast (IC). The IC is a measure of whether a joint intervention on two exposures has a greater additive association with incident HIV infection than expected based on the sum of the two individual interventions alone. The IC is 0 when there is no interaction.

## Results

A total of 2328 AGYW were HIV negative at enrollment and had at least one follow-up visit. The simulated cohort closely replicated the characteristics of the observed cohort of 2328 (Table 1). In the observed data over the study period, 75.4% (N = 5028 person visits) of AGYW lived in a household that had received a CSG (79.7% at enrolment), 22.1% (N = 1470 person visits) perceived that their parent/guardian cared a lot or somewhat (5.9% a lot, 16.2% somewhat and 77.9% not at all), and 94.1% (N = 6398 person visits) had high attendance in school. At enrollment, 17.1% (N = 391) had ever experienced physical IPV, and 18.5% (N = 410) were depressed. Simulated HIV risk over the study period (5.4%) closely matched the observed data (5.8%) (Table 1; Appendix Fig. 2).

**Table 1:** Characteristics of young women aged 13 to 20 without prevalent HIV and with at least one follow-up visit in Agincourt, South Africa enrolled in HPTN 068

	Enrollment observed (N = 2328) N (%)	All visits (N = 6796) N (%)	Enrollment Simulated (N = 10,000) N (%)	All visits simulated (N = 37,240) N (%)
Young women’s age at baseline (year)				
Age 13–14	734 (31.53)	2524 (37.19)	3144 (31.43)	12,388 (33.27)
Age 15–16	996 (42.78)	2947 (43.43)	4334 (43.34)	16,402 (44.04)
Age 17–18	498 (21.39)	1105 (16.28)	2044 (20.44)	6864 (18.43)
Age 18–20	100 (4.3)	210 (3.09)	478 (4.78)	1586 (4.26)
Household wealth				
Low	587 (25.26)	1298 (19.29)	2497 (25.02)	7194 (19.33)
Middle to low	620 (26.68)	1807 (26.85)	2707 (27.12)	9753 (26.20)
Middle	569 (24.48)	1898 (28.20)	2465 (24.69)	10,414 (27.98)
High	548 (23.58)	1727 (25.66)	2313 (23.17)	9861 (26.49)
CCT randomization arm	1214 (52.15)	3542 (52.20)	5251 (52.51)	19,556 (52.51)
Double or single orphan	100 (4.35)	371 (5.50)	462 (4.68)	1708 (4.64)
Feels close to parent	1917 (82.84)	5506 (82.78)	8255 (83.01)	30,715 (82.94)
Perception that parent/guardian cares a lot or somewhat	510 (22.02)	1470 (22.10)	2264 (22.73)	8617 (23.37)
Household receiving any grants	2055 (88.27)	5727 (85.58)	8780 (87.80)	32,766 (87.99)
Receiving CSG for at least one child in household	1856 (79.73)	5028 (75.43)	7964 (79.64)	28,107 (75.48)
Ever experienced any physical IPV	391 (17.15)	1051 (15.82)	1696 (17.29)	6171 (16.91)
High attendance in school (≥ 80% school days)	2255 (95.99)	6398 (94.09)	9552 (95.92)	34,338 (93.15)
Children’s depression inventory score ≥ 7	410 (18.53)	1176 (18.21)	1828 (19.21)	6679 (18.87)

Missing data in observed at enrollment: asset N = 4; parent close N = 14; parent cares N = 12; HSV 3; orphan 27; pregnant 28; death N = 1; CSG N = 273; depression 115; IPV 48; school attendance 10; Missing in observed over all visits; close parent = 135; care parent N = 133; assets N = 56; orphan N = 37; HSV N = 7; pregnant N = 181; death N = 95; receiving any grant including CSG N = 94; CSG N = 94; depression 329; IPV N = 58; high school attendance N = 18

Table 2 shows the potential effects of each individual exposure alone on HIV incidence. At 4 years, the risk of HIV if all AGYW received a CSG at each visit was 4.9% compared to 8.4% if no one received a CSG for a risk difference (RD) of − 3.5% (95% Confidence Interval (CI) − 8.1%, 1.2% (Table 2)). The risk of HIV if all AGYW received the CCT at enrollment was − 2.1% compared to none receiving the CCT (95% CI − 7.3%, 3.1%). Next, we compared the risk of HIV given the observed distribution of each risk factor to the risk of HIV if all girls had their risk removed (Table 2). All reductions in HIV incidence under this comparison were smaller and were less than 1%. However, given the 5.6% incidence in this study a − 0.9% reduction as was seen with caregiver care still corresponds with a 16% relative reduction in HIV incidence (0.9/5.6 = 16%).

**Table 2:** The modeled exposure effect and population attributable effect of intervening on each individual exposure on incident HIV infection among South African AGYW enrolled in HPTN 068

	Child support grant*	Caregiver care*	No depression at enrollment	Never IPV at enrollment	High school attendance*	CCT intervention
All exposed versus all unexposed						
Risk all unexposed (%, 95%CI)	8.4 (5.3, 11.6)	6.8 (4.6, 9.0)	10.3 (6.2, 14.4)	6.2 (2.7, 9.7)	14.6 (8.1, 21.1)	7.2 (4.8, 9.6)
Risk all exposed (%, 95%CI)	4.9 (2.3, 7.7)	4.6 (2.2, 7.0)	5.2 (3.4, 7.1)	6.1 (6.2, 8.0)	5.1 (3.1, 7.1)	5.1 (1.2, 9.0)
RD (%)	− 3.5 (− 8.1, 1.2)	− 2.2 (− 4.8, 0.4)	**− 5.1 (− 8.7, − 1.5)**	− 0.1 (− 3.1, 2.9)	**− 9.5 (− 16.0, − 2.9)**	− 2.1 (− 7.3, 3.1)
RR	0.59 (0.27, 1.27)	0.68 (0.41, 1.13)	**0.51 (0.32, 0.81)**	0.98 (0.60, 1.61)	**0.35 (0.19, 0.65)**	0.71 (0.26, 1.94)
All exposed versus observed						
Risk under observed (%, 95%CI)	5.6 (3.5, 7.6)	5.6 (3.5, 7.6)	5.6 (3.5, 7.6)	5.6 (3.5, 7.6)	5.6 (3.5, 7.6)	5.6 (3.5, 7.6)
Risk all exposed (%, 95%CI)	5.0 (2.3, 7.7)	4.6 (2.2, 7.0)	5.2 (3.4, 7.1)	6.1 (4.2, 8.0)	5.1 (3.1, 7.1)	5.1 (1.2, 9.0)
RD (%)	− 0.6 (− 1.9, 0.8)	− 0.9 (− 2.9, 1.1)	− 0.3 (− 1.3, 0.7)	0.5 (− 0.3, 1.4)	− 0.4 (− 1.2, 0.4)	− 0.4 (− 1.2, 0.4)
RR	0.90 (0.67, 1.20)	0.68 (0.41, 1.13)	0.95 (0.78, 1.15)	1.09 (0.93, 1.28)	0.92 (0.78, 1.09)	0.92 (0.51,1.66)

Bold if confidence intervals for risk differences do not cross null value of 0 and for risk ratios do not cross null value of 1

*Time-varying; measured as each visit

Table 3 shows the potential effects of reducing exposure to each risk factor paired with receipt of a CSG. Reducing exposure to each risk factor in combination with the CSG showed a greater reduction in HIV risk than reducing exposure to each risk factor alone. Receipt of a CSG plus caregiver care had the largest association with incident HIV infection when combining the CSG with a single intervention. At 4 years, the observed risk of HIV was 5.6% compared to 2.9% if all AGYW received a CSG at each visit and had caregiver care at each visit for a risk difference of − 2.6% (95% CI − 4.7%, − 0.6%). We found an interaction between the CSG and caregiver care (interaction contrast (IC) − 5.6%) indicating that the protective association with HIV became stronger with joint exposure. We did not find an additive interaction of the CSG with IPV or adolescent depression but did find a small interaction (IC = − 1.7%) with exposure to the CCT. For school attendance, we found that exposure to school attendance and the CSG actually had less of a protective relationship with HIV compared to what we would expect with each factor alone (IC was positive, 6.9%). This may be because each one is so strongly protective, and/or the two factors operate on a similar pathway, such that adding one to the other does not provide much additional protection.

**Table 3:** Risk ratios (RR), risk differences (RD; %) and 95% confidence intervals (CI) for the potential effect of various interventions on receipt of a child support grant (CSG) at each visit paired with caregiver care at each visit, eliminating adolescent depression at enrollment, eliminating IPV at enrollment, high attendance in school at each visit, or receipt of the CCT intervention at enrollment on incident HIV infection at 4 years of follow up among South Africa AGYW enrolled in HPTN 068

	CSG plus Caregiver care	CSG plus No depression	CSG plus No IPV	CSG plus High school attendance	CSG plus CCT
Total effect					
Risk under observed (%, 95%CI)	5.6 (3.5, 7.6)	5.6 (3.5, 7.6)	5.6 (3.5, 7.6)	5.6 (3.5, 7.6)	5.6 (3.5, 7.6)
Risk under all exposed (%, 95%CI)	2.9 (0.2, 5.6)	3.8 (1.5, 6.1)	4.5 (1.9, 7.2)	4.2 (1.6, 6.9)	3.9 (− 0.9, 8.7)
RD (%)	**− 2.6 (− 4.7, − 0.6)**	**− 1.7 (− 3.0, − 0.4)**	− 1.0 (− 2.4, 0.4)	− 1.3(− 2.7, 0.1)	− 1.7 (− 5.1, 1.8)
RR	**0.52 (0.29, 0.96)**	**0.69 (0.49, 0.97)**	0.82 (0.59, 1.14)	0.76 (0.55, 1.07)	0.70 (0.2, 2.1)
Interaction contrast (IC)*	− 5.25%	− 0.32%	0.19%	6.85%	− 1.67%

Bold if confidence intervals for risk differences do not cross null value of 0 and for risk ratios do not cross null value of 1

*IC < 0 indicates a net increase in the inverse effect with joint exposure (synergism; IC > 0 indicates a net reduction in the inverse effects with joint exposure (antagonism). IC = 0 if R_11_−R_00_ = (R_10_−R_00_) + (R_01_−R_00_) [60]l

**Fig. 1:**
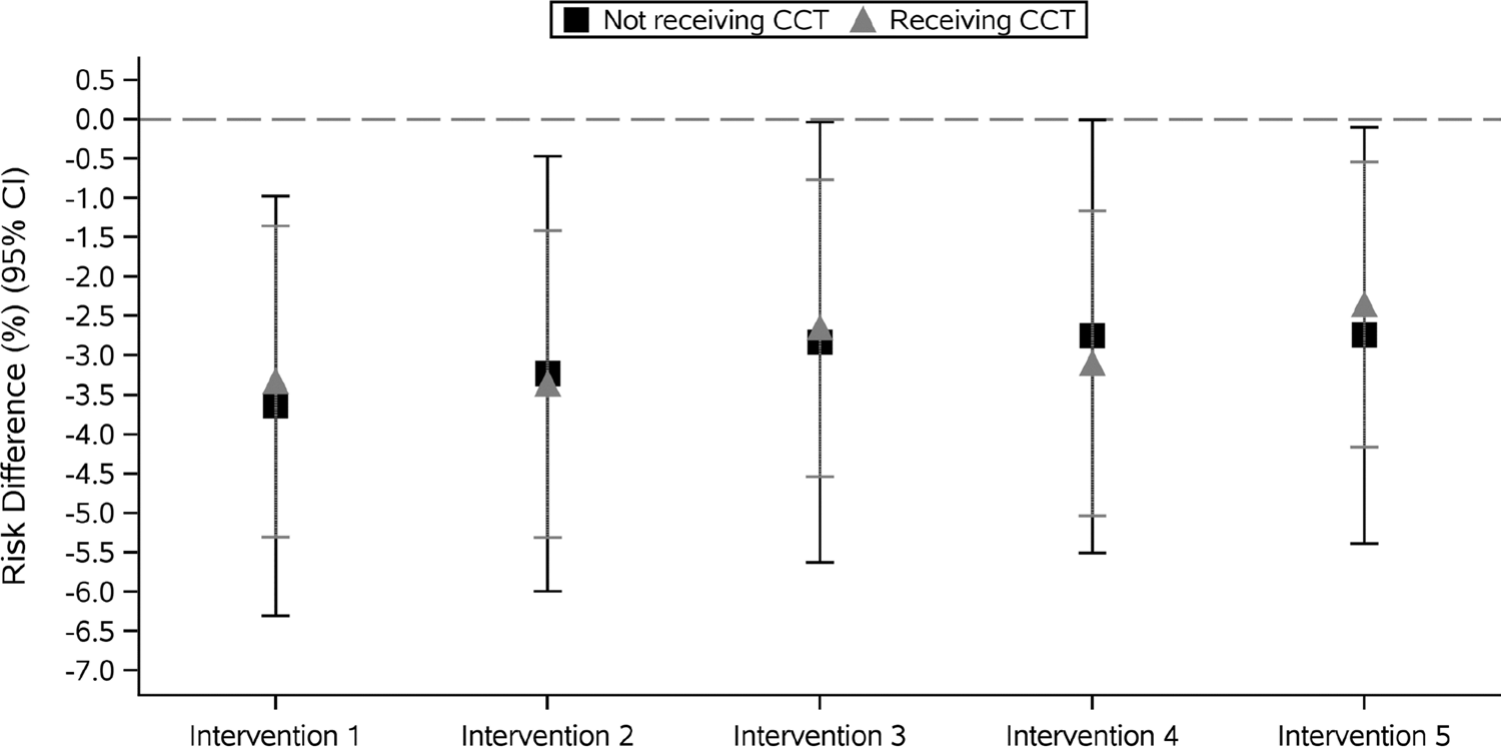
Risk differences (RD; %) and 95% confidence intervals (CI) for the potential effect of various interventions on a combination of receipt of a child support grant (CSG) at each visit, increasing caregiver care at each visit, eliminating adolescent depression at enrollment, eliminating IPV at enrollment and high attendance in school at each visit on incident HIV infection at 4 years of follow up, stratified by receipt of conditional cash transfer (CCT) intervention. *Intervention 1* Provide CSG, increase caregiver care, eliminate adolescent depression, eliminate IPV, and increase school attendance. *Intervention 2* Provide CSG, eliminate adolescent depression and increase caregiver care; *Intervention 3* Provide CSG, eliminate IPV and increase caregiver care; *Intervention 4* Provide CSG, increase caregiver care and increase school attendance. *Intervention 5* Intervention to provide CSG and increase caregiver care

Table 4 examines all combinations of interventions on risk factors plus receipt of a CSG. The combinations that included receipt of the CSG and caregiver care had the largest association with incident HIV (Interventions 1–4 and 8–12). Intervention 1 to provide a CSG at each visit, increase caregiver care at each visit, eliminate adolescent depression at enrollment, eliminate IPV at enrollment, and increase school attendance at each visit had the largest reduction in risk of HIV at 4 years (RD − 3.4%; 95% CI − 5.5, − 1.4). Intervention 2 showed a similar reduction in HIV risk at 4 years as intervention 1 and did not require an intervention on school attendance or IPV (RD − 3.0%; (95% CI − 5.1, − 0.9). Interventions 8–12 with the addition of the CCT showed similar reductions in HIV incidence to interventions 1–4 without the CCT. Figure 1 shows the interventions with the largest potential effects on HIV incidence from Table 4, stratified by receipt of the CCT. There were no substantial differences in the associations of any potential interventions with HIV when stratified by receipt of the conditional cash transfer intervention (Fig. 1).

**Table 4:** Risk differences (RD; %) and 95% confidence intervals (CI) for the potential effect of various interventions on a combination of receipt of a child support grant (CSG) at each visit, caregiver care at each visit, eliminating adolescent depression at baseline, eliminating IPV at baseline, high attendance in school at each visit and receipt of CCT intervention at enrollment on incident HIV infection at 4 years of follow up among South Africa AGYW enrolled in HPTN 068

Intervention	Risk under all exposed (95%CI)	Risk under observed (95% CI)	Risk difference (95% CI)*
(1) CSG, increase caregiver care, eliminate adolescent depression, eliminate IPV, and increase school attendance	2.1 (− 0.2, 4.4)	5.6 (3.5, 7.6)	**− 3.4 (− 5.5, − 1.4)**
(2) CSG, eliminate adolescent depression and increase caregiver care	2.5 (0.1, 5.0)	5.6 (3.5, 7.6)	**− 3.0 (− 5.1, − 0.9)**
(3) CSG, increase caregiver care and increase school attendance	3.2 (0.5, 6.0)	5.6 (3.5, 7.6)	**− 2.3 (− 4.5, − 0.2)**
(4) CSG, eliminate IPV and increase caregiver care	3.4 (0.6, 6.1)	5.6 (3.5, 7.6)	− 2.2 (− 4.4, 0.0)
(5) CSG, eliminate adolescent depression, and increase school attendance	3.5 (1.2, 5.8)	5.6 (3.5, 7.6)	**− 2.0 (− 3.4, − 0.7)**
(6) CSG, eliminate adolescent depression and eliminate IPV	3.6 (1.3, 6.0)	5.6 (3.5, 7.6)	**− 1.9 (− 3.3, − 0.5)**
(7) CSG, eliminate IPV, and increase school attendance	4.0 (1.5, 6.4)	5.6 (3.5, 7.6)	**− 1.6 (− 2.9, − 0.2)**
Interventions from above plus CCT intervention			
(8) CCT, CSG, increase caregiver care, eliminate adolescent depression, eliminate IPV, and increase school attendance	1.7 (− 1.5, 4.9)	5.6 (3.5, 7.6)	**− 3.9 (− 6.3, − 1.5)**
(9) CCT, CSG, increase caregiver care and increase school attendance	2.2 (− 1.8, 6.2)	5.6 (3.5, 7.6)	**− 3.4 (− 6.4, − 0.4)**
(10) CCT, CSG, eliminate adolescent depression and increase caregiver care	1.9 (− 1.5, 5.3)	5.6 (3.5, 7.6)	**− 3.6 (− 6.2, − 1.1)**
(11) CCT, CSG, eliminate IPV and increase caregiver care	2.6 (− 1.4, 6.6)	5.6 (3.5, 7.6)	− 2.9 (− 5.9, 0.1)
(12) CCT, CSG and increase caregiver care	2.9 (− 1.2, 7.1)	5.6 (3.5, 7.6)	− 2.6 (− 5.7, 0.4)

*Bold if confidence intervals for risk differences do not cross null value of 0

## Discussion

Our analysis demonstrates that combining a government cash transfer program with other interventions to address psychosocial factors may substantially improve the impact of such a program on HIV incidence. Neither the CSG nor the CCT intervention were independently associated with HIV incidence. When examining the impact of the CSG paired with intervening on other risk factors, receipt of the CSG plus caregiver care showed by far the largest decrease in incident HIV infection. The relationship of the CSG plus parental care on HIV incidence was further improved by reducing exposure to all other risk factors. A potential intervention to provide a CSG, decrease adolescent depression, and increase caregiver care had the largest reduction in HIV incidence with changes in the fewest number of risk factors. Reductions in HIV incidence were not substantially increased by adding receipt of the CCT intervention or modified by the CCT intervention. Therefore, receipt of the CCT may not have provided additional benefit over the CSG alone or in combination with other interventions.

Our findings support the rationale for cash interventions in tandem with other interventions to increase psychosocial support. Prior work by Cluver et al. found that receipt of the South African CSG plus caregiver care or other psychosocial interventions results in a larger reduction in sexual risk behaviors than receipt of cash alone [14, 15]; our results support these findings and add to them by showing potential effects on HIV incidence. We find that the protective association of the CSG was stronger with joint exposure to caregiver care, and combinations including these two factors were associated with the largest reductions in HIV incidence compared to other combinations examined. Combination interventions may have an added impact on HIV because they address multiple mechanisms of risk, multilevel and intersecting social factors and reduce cumulative experiences of adversity [24]. Prior findings also suggest that social protection may act as the ‘glue’ for cash programs to have positive effects, or vice versa, that combined interventions bolster social pathways associated with improved resilience and that they may facilitate access to services for vulnerable populations [14, 15]. Therefore, cash plus care interventions in our study may have had more of an association over cash alone because of increases in social support that improve mental health or facilitate access to cash, health services, or other resources like school that protect against HIV. We add to prior evidence by demonstrating that cash plus care can impact HIV incidence and by examining the combined effect of intervening on multiple structural and behavioral factors simultaneously. Additionally, while we know that combined strategies for HIV prevention can be effective, these programs are often very large and resource intensive. Our analysis helps to identify which specific interventions could be combined to reduce the most infections from a large list of possible combinations.

Caregiver care emerged as the most important factor to reduce HIV incidence in AGYW in combination with the CSG. Caregiver care in combination with the CSG showed larger reductions in HIV incidence than receipt of the CSG alone. This greater reduction in risk may reflect the fact that government grants are given at the household level and therefore may benefit young women more in households where they have a supportive parent/caregiver who includes the needs of the AGYW in household decision-making. There may also be other mechanisms through which the combined effects of cash plus care operate such as improved mental health, social support, and overall well-being of the child. Adolescent depression emerged as another key factor for intervention to reduce HIV incidence. A recent systematic review of mental health interventions found that universally delivered interventions can improve adolescent mental health and reduce risk behavior, particularly those that include interpersonal skills training, emotional regulation, and alcohol and drug education [53].

While the percentage of AGYW that reported they had a parent/caregiver that cared a lot or somewhat was low (less than a quarter), we found it to be highly associated with lower risk of HIV infection. This evidence highlights the importance of strengthening caregiver-child relationships to prevent HIV among AGYW. Family based HIV-prevention programs have increased safer sexual behaviors and improved adherence and other behaviors in HIV positive youth [54–56]. Yet, research is still needed to fully understand how families can be supported and encouraged to promote healthy behaviors in young women and reduce risk of HIV [57].

There are several limitations to our analysis. First, the g-formula relies on the assumptions of correctly specified models and no unmeasured confounding [44]. We saw that our simulated cohort was similar to the observed data in HIV risk and other characteristics, suggesting that the models were adequately specified. We also assumed causal consistency (that any differences between individuals in mechanism of exposure assignment are ignorable), positivity (we have individuals exposed and unexposed in all covariate strata) and that the exposure precedes the outcome (we used HIV infections that occurred at the next time point after exposure to ensure temporality). We ensured that the assumption of temporality and positivity were met through sensitivity analyses, however, it is possible that participants may have interpreted the question about caregiver care differently, therefore violating our assumption of consistency.

Additionally, our analysis relies on self-reported information and sexual behaviors that may be misreported due to social desirability bias and our data come from a randomized controlled trial of a CCT intervention. Prior analyses of this data have shown that there was selection bias and a Hawthorne effect where girls in the study were more likely to be enrolled in school and stay in school than the underlying population in the study area [58]. However, if the prevalence of school dropout were higher, as we would expect in the larger underlying population, then changes in school attendance and other related characteristics would have more of an effect because reductions in prevalence would be larger [59].

In conclusion, addressing social determinants such as poverty through the use of cash transfer programs can have an impact on HIV incidence in AGYW when combined with other social and behavioral factors such as caregiver care and mental health services. Our results support the need for cash plus other structural and psychosocial programs. Household cash transfers could be combined with interventions to improve caregiver care and reduce adolescent depression to more effectively reduce HIV incidence among adolescent girls and young women.

## Figures and Tables

**Fig. 2:**
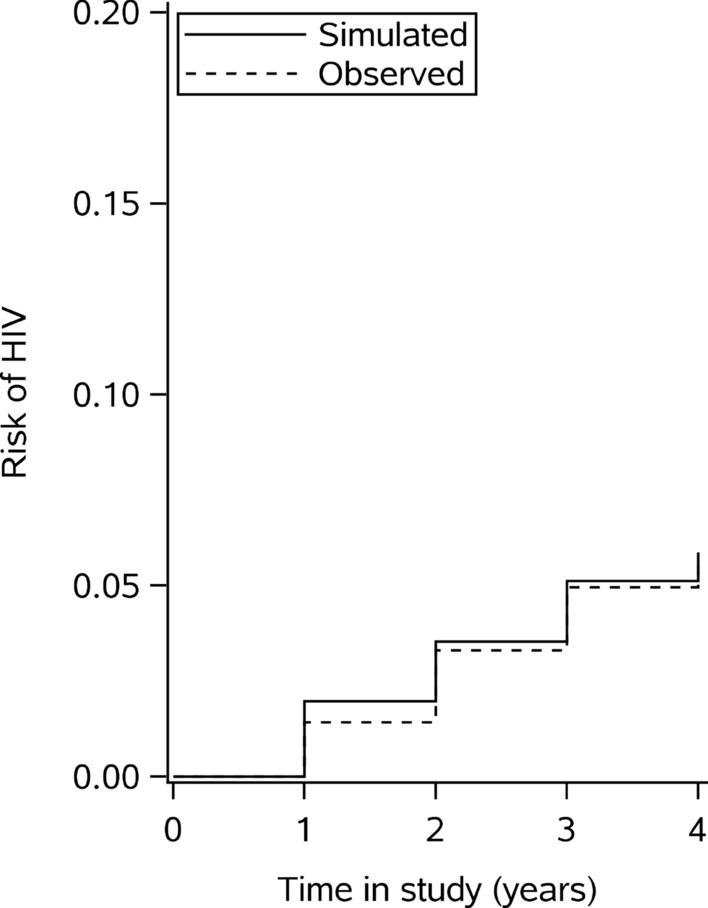
Observed versus simulated cumulative incidence of HIV by time since study enrollment

## Data Availability

Data from the HPTN 068 study are currently available through the HIV Prevention Trials Network.

## Appendix

See Fig. 2.

## Code Availability

Code for this project is available by contacting the corresponding author.
