# Correction to: Modeling Cash Plus Other Psychosocial and Structural Interventions to Prevent HIV Among Adolescent Girls and Young Women in South Africa (HPTN 068)

**DOI:** 10.1007/s10461-021-03261-5

**Published:** 2021-04-22

**Authors:** Marie C. D. Stoner, Jessie K. Edwards, Daniel Westreich, Kelly Kilburn, Jennifer Ahern, Sheri A. Lippman, F. Xavier Gómez-Olivé, Kathleen Kahn, Audrey Pettifor

**Affiliations:** 1 Women’s Global Health Imperative, RTI International, Berkeley, CA USA; 2 Carolina Population Center, University of North Carolina, Chapel Hill, NC USA; 3 Department of Epidemiology, University of North Carolina, Chapel Hill, NY USA; 4 MRC/Wits Rural Public Health and Health Transitions Research Unit (Agincourt), School of Public Health, Faculty of Health Sciences, University of the Witwatersrand, Johannesburg, South Africa; 5 INDEPTH Network, Accra, Ghana; 6 Epidemiology and Global Health Unit, Department of Public Health and Clinical Medicine, Umeå University, Umeå, Sweden; 7 Department of Medicine, University of California, San Francisco, USA; 8 Division of Epidemiology & Biostatistics, School of Public Health, University of California, Berkeley, USA; 9 Duke Global Health Institute, Duke University, Durham, NC USA

## Correction to: AIDS and Behavior 10.1007/s10461-021-03158-3

The article “Modeling Cash Plus Other Psychosocial and Structural Interventions to Prevent HIV Among Adolescent Girls and Young Women in South Africa (HPTN 068)”, written by Marie C. D. Stoner, Jessie K. Edwards, Daniel Westreich, Kelly Kilburn, Jennifer Ahern, Sheri A. Lippman, F. Xavier Gómez-Olivé, Kathleen Kahn, Audrey Pettifor was originally published electronically on the publisher’s internet portal on 21st January 2021 without open access. With the author(s)’ decision to opt for Open Choice the copyright of the article changed on 9th April 2021 to © The Author’s 2021 and the article is forthwith distributed under a Creative Commons Attribution.

“The original article has been corrected.”

